# Treatment of a Diseased Tooth

**Published:** 1860-08

**Authors:** 


### TREATMENT OF A DISEASED TOOTH.

BY J. T.

Mr. G. D. P., aged 28 years, of nervous sanguine temperament, and in good health; had the second right inferior bicuspid filled in its anterior approximal surface with "os artificial," about two weeks previous to this examination.

For a few days after it was filled, there was no pain except upon taking co'd water into the mouth; but within a week the tooth has become quite sore from inflammation of the periostium, and ached almost constantly.

The filling was a very poor one and badly put in, a portion of it being already displaced, and the remainder quite soft, upon removing it, the pulp was found to be exposed, and partially paralized. Enlarging the orifice of exposure, the pulp was easily removed from the whole length of the fang. Cleaned out the fang, applied creosote, and let it remain six hours, at which time the soreness of the tooth was entirely gone. Indeed, it was almost entirely gone immediately after the removal of the pulp. This, I suppose, was brought about by the hemorrhage consequent upon the removal of the pulp. Then filled the fang and decayed cavity with gold. The operation caused no pain, nor was there any soreness of the tooth after it was filled, and all is well to the present time, which is about one month.

Before filling, placed a small pledget of cotton moistened with creosote, in the canal near the apex of the fang, and filled down upon it firmly.

Applied tincture of iodine to the gums immediately after removing the pulp, and at the close of the operation.

This case illustrates the fact that oftentimes success is better attained by prompt, energetic treatment than by delaying for more mild treatment. The difficulty in the periostium was removed by the hemorrhage, after the removal of the pulp, whereas, had arsenic been employed, far greater difficulty would have been experienced with the periostial irritation. Cases of an acute character will often result best with prompt, thorough treatment at once.

